# Identification of Upregulated HNRNPs Associated with Poor Prognosis in Pancreatic Cancer

**DOI:** 10.1155/2019/5134050

**Published:** 2019-07-04

**Authors:** Lu Qiao, Ning Xie, Yuru Bai, Yan Li, Yongquan Shi, Jinhai Wang, Na Liu

**Affiliations:** ^1^Department of Gastroenterology, The Second Affiliated Hospital of Xi'an Jiaotong University, Xi'an, Shaanxi Province, China; ^2^Shaanxi Key Laboratory of Gastrointestinal Motility Disorders, The Second Affiliated Hospital of Xi'an Jiaotong University, Xi'an, Shaanxi Province, China; ^3^Department of Geriatric Respiratory and Endocrinology (The Third Unit of Cadre's Ward), The Second Affiliated Hospital of Xi'an Jiaotong University, Xi'an, Shaanxi Province, China; ^4^State Key Laboratory of Cancer Biology, The Fourth Military Medical University, Xi'an, Shaanxi Province, China; ^5^Xijing Hospital of Digestive Diseases, Xijing Hospital, The Fourth Military Medical University, Xi'an, Shaanxi Province, China

## Abstract

Heterogeneous nuclear ribonucleoproteins (HNRNPs) are reported to play a crucial role in the pathogenic process of multiple malignancies. However, the expression patterns and prognostic values of HNRNPs in pancreatic cancer (PC) are lacking. In this study, several public databases were explored to identify the commonly upregulated HNRNPs in PC. The clinical significance of HNRNPL (heterogeneous nuclear ribonucleoproteins L) in PC was analyzed. We further performed a series of experiments to elucidate the biological functions of HNRNPL. Bioinformatics analysis including pathway enrichment and interactors with HNRNPL was used to explain the potential mechanisms of HNRNPL in PC pathogenesis. Herein, we reported that HNRNPL was commonly overexpressed in public databases and that high expression of HNRNPL in PC was positively associated with aggressive disease and poor overall survival. Downregulation of HNRNPL suppressed the abilities of migration and epithelial mesenchymal transition of PC cells in vitro, while depletion of HNRNPL did not affect the proliferation rate of PC cells. We further showed that HNRNPL might combine with RNA-binding protein, PTBP1, and function as a part of the spliceosome to regulate alternative splicing of target genes in the occurrence and development of PC. HNRNPL could be employed as an innovative prognostic biomarker and therapeutic target for PC.

## 1. Introduction

Pancreatic cancer (PC) is one of the most common malignancies worldwide and the fourth leading cause of cancer-related deaths in USA with an estimated 55,440 cases and 44,330 deaths per year [1], and it is projected to surpass breast cancer to become the second leading cause of cancer-related death in decades [2]. Due to a lack of nonspecific symptoms at early stage, the great majority of PC patients are diagnosed with advanced-stage disease, issuing in extremely low five-year survival rates [3, 4]. Although lots of researches focusing on pancreatic cancer have been done, the early diagnostic rates and five-year survival rates are still unsatisfied [5]. Hence, it is significant and urgent to identify effective prognostic indicators and new therapeutic targets for pancreatic cancer.

Heterogeneous nuclear ribonucleoproteins (HNRNPs), as key members of the RNA-binding proteins (RBPs), were proven to function as regulators of alternative splicing, linking the premessenger RNA (pre-mRNA) to the splicing machinery [6]. Recently, HNRNPs have been implicated in multiple aspects of the occurrence and development of tumors [6, 7]. HNRNPM was found to be upregulated in breast cancer, and it could promote breast cancer invasion and metastasis via regulating CD44 alternative splicing [8]. HNRNPK could regulate the epithelial mesenchymal transition (EMT) in non-small-cell lung cancer and modulate apoptosis in osteosarcoma [9]. HNRNPA1 protein was found overexpressed in lung cancer tissues [10]. HNRNPF was aberrantly high-expressed in two primary human Merkel cell carcinoma cell lines and tumor tissue microarray [11]. Several studies have reported that HNRNPs participate in the molecular mechanisms of PC [12–14], while few studies focused on the expression patterns and prognostic values in PC.

Herein, this manuscript took advantage of multiple public databases including The Cancer Genome Atlas (TCGA), Gene Expression Omnibus (GEO), and Oncomine databases to identify the commonly upregulated HNRNPs in PC. Additionally, HNRNPL was demonstrated to be an independent factor for overall survival (OS) and positively associated with advanced clinical stage of PC. Experiments in vitro were performed to discover that downregulation of HNRNPL could impede the migration ability and EMT process in PC cell lines, while it could not inhibit proliferation of pancreatic cancer cells. Moreover, public databases were explored to study potential molecular mechanisms of HNRNPL in pancreatic cancer.

## 2. Materials and Methods

### 2.1. Public Databases

#### 2.1.1. TCGA Dataset Analysis

GEPIA (http://gepia.cancer-pku.cn/) [15] was used to analyze all of the upregulated genes via ANOVA test in TCGA-PAAD (pancreatic adenocarcinoma). LogFC and* P* values were obtained from the website. All upregulated genes were selected as significant with the criterion of combined adjusted* P *< 0.001 and logFC > 1.5. The boxplot of* HNRNPL* in PC was downloaded from GEPIA. The Pearson correlation between* HNRNPL* and* PTBP1* was downloaded from the website.

The clinical information (March 2017) regarding TCGA-PAAD was downloaded from the website. We ultimately obtained 178 cases after excluding 1 case without clinical and pathological data, including 80 females and 98 males. Among 178 cases, 97 patients were older than 65 years old, and 81 patients were younger than 65 years old. 21 patients were diagnosed as TNM stage I, 146 were stage II, 4 were stage III, 5 were stage IV, and the stages of the rest of patients (2 patients) remained unclear. The median follow-up duration was 566.63 days (ranging from 0 to 2741 days).

#### 2.1.2. GEO

The GSE16515 [16] microarray data were obtained from GEO (http://www.ncbi.nlm.nih.gov/geo/). There were 36 pancreatic cancer tissues and 16 adjacent nontumor mucosa in GSE16515. GEO2R was used to identify all of the upregulated genes in tumor tissues as opposed to noncancerous tissues.

#### 2.1.3. Oncomine Database Analysis

Oncomine (http://www.oncomine.org) [17] was utilized to examine the mRNA expression difference of* HNRNPL *between tumor and normal tissues. We computed the average of expression levels of different probes of HNRNPL. A t-test was examined to calculate the significance between tumor and normal tissues of the pancreas from Segara Pancreas [18].

### 2.2. Clinical Samples

The pancreatic cancer tissue microarray was purchased from Shanghai Outdo Biotech: HPanA150CS03, which contains 80 distinct pancreatic cancer tissues, determined by HE staining.

### 2.3. Immunohistochemistry (IHC)

The avidin-biotin complex immunoperoxidase method was reported in a previous study [19]. The section was incubated with monoclonal mouse anti-HNRNPL (sc-32317, Santa, USA) at 1:200 dilution. According to staining intensity in the majority of specimens, immunoreactivity was scored as absent (−), weak (+), moderate (++), or strong (+++) [19].

### 2.4. Cell Culture

Pancreatic ductal adenocarcinoma cell lines, PATU8988T, SW1990, and BXPC-3 (Cell bank of Chinese Academy of Sciences, Shanghai, China), were kept at 37°C and 5% CO_2_ in Roswell Park Memorial Institute (RPMI)-1640 (HyClone, USA) supplemented with 10% fetal bovine serum (FBS, Gibco, Grand Island, NY).

### 2.5. Cell Transfection

The shRNA targeting human HNRNPL (5′- CACUGGUGGAGUUUGAAGATT -3′) and the negative control (NC) shRNA (5′-TTCTCCGAACGTGTCACGT-3′) were cloned into the GV493 vector (GeneChem, Shanghai, China) carrying the puromycin resistance gene. Transfected PATU8988T cells were selected in 5 *μ*g/mL puromycin (Solarbio, Beijing, China) and SW1990 and BXPC-3 were selected in 2.5 *μ*g/mL puromycin for 2 weeks. The efficiency of knockdown was confirmed using Western blot detection.

### 2.6. Cell Proliferation Assay

The CCK8 assay (Dojindo, Japan) was used to measure the proliferation abilities of different cell lines according to the manufacturer's introductions. 1 x 10^3^ cells from PATU8988T and 2 x 10^3^ cells from SW1990 were plated in 100*μ*L medium in 96 well plates in five replicates. Cells were incubated in 10% CCK-8 which was diluted in normal culture medium at 37°C for 2 hours. Proliferation rates were determined at 0, 24, 48, 72, and 96 hours after plating.

### 2.7. Migration Assays

Transwell chamber migration assays (Corning, NY, USA) were used to determine the respective migratory capacity. 4 x 10^5^ PATU8988T cells and 5 x 10^5^ BXPC-3 cells were resuspended in FBS-free medium and plated into the upper chambers, while the lower chambers were loaded with medium containing 10% FBS. The PATU8988T cells were incubated for 24 hours, and the BXPC-3 cells were incubated for 96 hours. The quantification was performed under a microscope using 200x magnification.

### 2.8. Western Blot

The specific protocol was reported in a previous study [19]. The blots were stained with anti-HNRNPL Ab (diluted 1:1000; Santa Cruz, USA), anti-N-cadherin (1:1000, CST, USA), anti-E-cadherin (1:1000, CST, USA), and anti-GAPDH monoclonal Ab (1:2000, Proteintech, Chicago, USA), followed by incubation with species-specific secondary antibodies. Enhanced chemiluminescence (Millipore, USA) was used for detection.

### 2.9. Network Analysis

All cases in TCGA-PAAD were divided into two groups by* HNRNPL* median expression:* HNRNPL* high expression and* HNRNPL* low expression groups. Next, Gene Set Enrichment Analysis (GSEA) was performed to determine which pathway HNRNPL was involved in. Protein-protein interaction (PPI) genes with HNRNPL were obtained from the BioGRID database (https://thebiogrid.org/) [20]; interaction genes supporting with at least 2 evidence were present.

### 2.10. Statistical Analyses

All the patients were divided into high and low expression groups according to the median expression of* HNRNPA2B1* or* HNRNPL*. Survival probabilities were estimated using the Kaplan-Meier method and the log-rank test. The correlation between HNRNPL protein expression and clinicopathologic features was analyzed with the rank sum test. Difference between two groups was analyzed by Mann-Whitney U test, while Kruskal-Wallis H test was used when more than two groups. All experiments were repeated at least three times. Venn diagrams were generated by Venn Diagram Plotter.* P* values less than 0.05 were considered significant (*∗ P* < 0.05, *∗∗ P* < 0.01, *∗∗∗ P* < 0.001, and *∗∗∗∗ P* < 0.0001).

## 3. Results

### 3.1. Identification of Upregulated HNRNPs in PC

**Figure 1:**
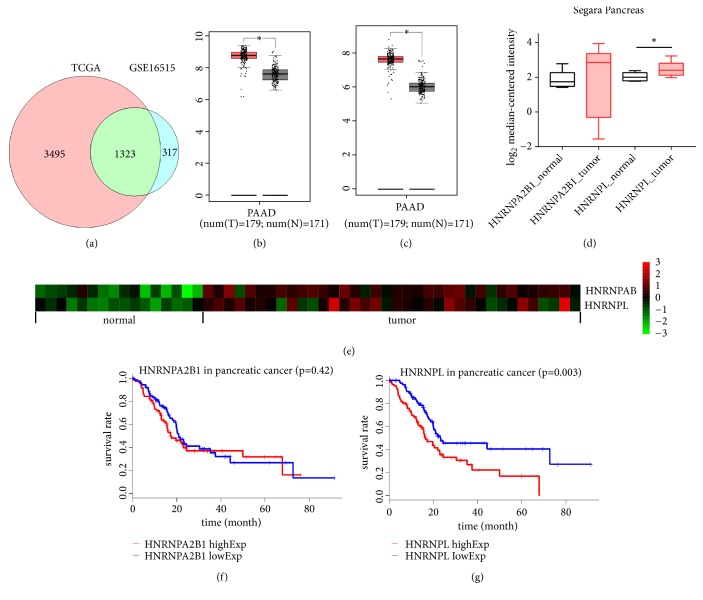
Expression patterns and prognostic values of HNRNPs in pancreatic cancer. (a) Venn diagram of upregulated genes in pancreatic cancer from TCGA and GSE16515. (b) The expression patterns of* HNRNPA2B1* and (c)* HNRNPL* in TCGA (T: tumor in red, N: normal in grey, *∗ P* < 0.05). (d) The mRNA levels of* HNRNPA2B1* and* HNRNPL* in Segara Pancreas and (e) GSE16515 (*∗ P* < 0.05). (f-g) The prognostic values of* HNRNPA2B1* and* HNRNPL* in TCGA-PAAD.

To explore the overexpressed HNRNPs in PC, all upregulated genes in pancreatic cancer versus normal tissues from TCGA-PAAD and GSE16515 were determined. 4818 upregulated genes (logFC > 1.5,* P* value < 0.001) were found in TCGA-PAAD, while 1640 overexpressed genes (logFC > 0.45,* P* value < 0.001) were found in GSE16515. Venn diagram analysis showed that there were only 2 HNRNPs among the commonly upregulated genes in TCGA and GSE16515 and that they were* HNRNPL* and* HNRNPA2B1 *(Figure 1(a)). The mRNA levels of* HNRNPL *and* HNRNPA2B1* in GEPIA and GSE16515 were illustrated in Figures 1(b), 1(c), and 1(e). To further test the expression patterns of* HNRNPL* and* HNRNPA2B1* in PC, we employed one Oncomine dataset and found that* HNRNPL* was statistically higher in pancreatic cancer as opposed to normal tissues, while* HNRNPA2B1* was not significantly upregulated in Segara Pancreas (Figure 1(d)). Thus, we concluded that* HNRNPL* was commonly overexpressed in pancreatic tumor tissues compared with noncancerous tissues in multiple public databases.

### 3.2. The Prognostic Values and Clinical Significance of HNRNPL and HNRNPA2B1 in PC Patients

To recognize the roles of HNRNPL and HNRNPA2B1 in PC, we studied the prognostic values of* HNRNPL* and* HNRNPA2B1* in TCGA-PAAD. Kaplan-Meier analysis illustrated that higher expressed* HNRNPL* was correlated with shorter OS of PC patients (23.17 vs. 16.60 months;* P* = 0.003, Figure 1(g)), while the level of* HNRNPA2B1* did not affect PC patients' survival (23.13 vs. 17.27 months;* P* = 0.42, Figure 1(f)). To clarify the prognostic value of* HNRNPL*, Cox regression multivariate analysis was performed, and the results indicated that* HNRNPL* was an independent prognostic factor for the OS of PC, which it was independent of tumor size, TNM stage, and histologic grade (Table 1). Furthermore, we examined the correlation between* HNRNPL* levels and clinicopathological data of PC patients (Table 2). The results illustrated that* HNRNPL* was correlated with gender, tumor invasion depth, and TNM stage. Male PC patients tended to have higher* HNRNPL *levels. The higher the* HNRNPL*, the deeper the PC invaded and the higher the TNM stage of neoplasm. Thus, we reached the tentative conclusion that HNRNPL was the key molecule which played a fundamental part in PC among the HNRNP family.

### 3.3. Protein Levels of HNRNPL in PC and Its Association with the Clinicopathological Features of PC Patients

**Figure 2:**
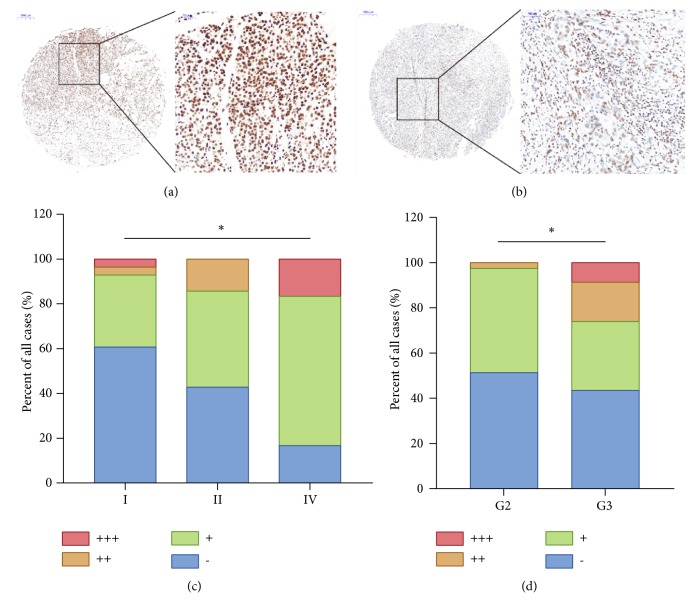
The expression pattern and clinical significance of HNRNPL protein levels in PC. (a), (b) Examples of IHC staining of HNRNPL. (a) PDAC tissues with strong positive HNRNPL staining (left, 40×, right 200×). (b) PDAC tissues with mid-strong positive HNRNPL staining (left, 40×, right 200×). (c) HNRNPL protein expression was positively correlated with tumor TNM stages and (d) pathologic grades (*∗ P* < 0.05).

With the aim of studying the protein level of HNRNPL in PC, one tissue microarray was employed and IHC staining showed that HNRNPL was mostly expressed in the nucleus of pancreatic cancer cells (Figures 2(a)-2(b)), which implied that HNRNPL might function similarly to other RBPs and participate in RNA splicing and metabolism. We further tested the correlation of HNRNPL protein expression and clinical pathologic data and suggested that HNRNPL was associated with tumor invasion of PC (Table 3). The higher the HNRNPL, the deeper the PC invaded (Figure 2(c)), which agreed with the HNRNPL mRNA levels in the previous section of the manuscript. HNRNPL was also correlated with pathological grade, and the higher the HNRNPL expressed, the higher the pathological grade of the neoplasm (Figure 2(d)).

### 3.4. Downregulation of HNRNPL Inhibits Pancreatic Cancer Cell Lines Migration through Regulating EMT

**Figure 3:**
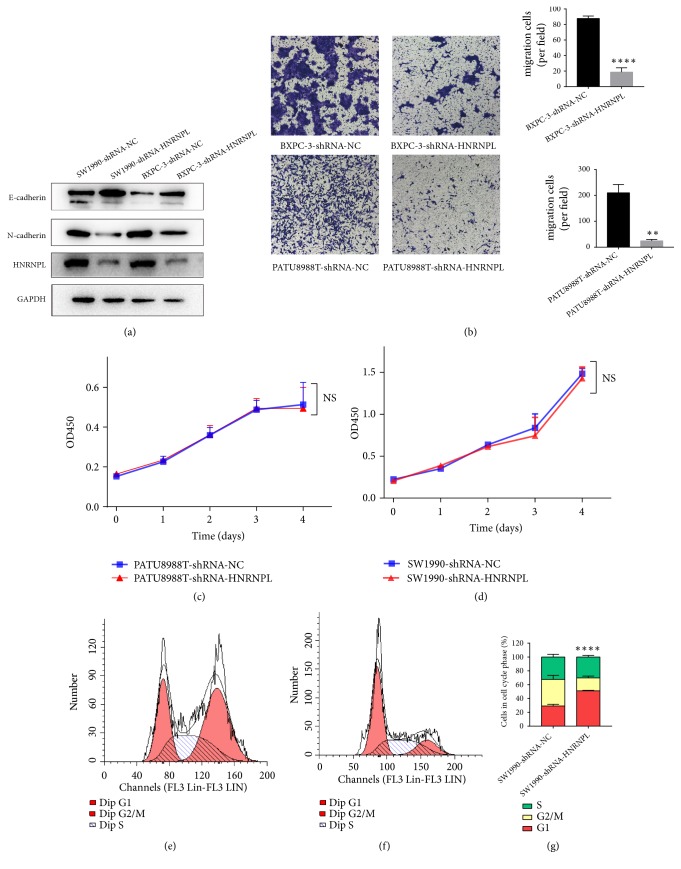
Downregulation of HNRNPL impairs cell migration and regulates expression of EMT-related proteins. (a) Western blot analysis of EMT markers in SW1990 and BXPC-3 pancreatic cancer cell lines (shRNA-NC vs shRNA-HNRNPL). (b) Transwell migration assays showing different migration abilities of PC cell lines (BXPC-3 and PATU8988T, *∗∗∗ P* <0.001, *∗∗∗∗ P* <0.0001). (c-d) Downregulation of HNRNPL does not affect proliferation of PATU8988T and SW1990. (e-g) Downregulation of HNRNPL regulates cell cycle arrest in SW1990 cell lines (*∗∗∗∗ P* <0.0001).

Transwell migration assays showed that the migration rate of negative control cells was greater than that PATU8988T and BXPC-3 cells depleted of HNRNPL by shRNA (Figure 3(b)), indicating that HNRNPL deficiency impaired the migration ability of pancreatic cancer cells. Additionally, Western blot data revealed that BXPC-3 and SW1990 cells transfected with shRNA expressed high levels of E-cadherin (Figure 3(a)). Knockdown of HNRNPL decreased the expression of mesenchymal biomarkers of N-cadherin (Figure 3(a)). Altogether, these results strongly demonstrate that HNRNPL promotes the invasiveness of PC through EMT processes.

### 3.5. The Impact of HNRNPL on Pancreatic Cancer Cell Proliferation and Cell Cycle

We investigated the role of HNRNPL on the proliferation ability of PC cells in vitro. The CCK-8 assay results demonstrated that HNRNPL downregulation did not alter the proliferation of PATU8988T and SW1990 cell lines (Figures 3(c)-3(d)).

Based on these results, we investigated the effect of HNRNPL expression on the pancreatic cancer cell cycle. Cell cycle analysis revealed that downregulation of HNRNPL in SW1990 cells reduced the percentage of cells entering S phase and caused an accumulation of cells in G1, relative to negative control cells (Figures 3(e)–3(g)), while there was no obvious difference in PATU8988T cells.

### 3.6. The Potential Pathways of HNRNPL in the Development of PC

**Figure 4:**
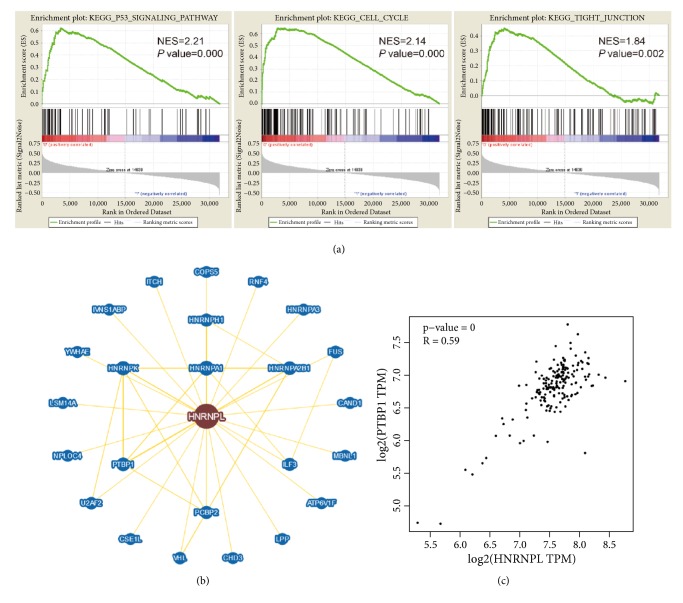
The potential pathways of HNRNPL in the development of PC. (a) GSEA analysis of HNRNPL in TCGA. (b) Network of HNRNPL with its interactors in Homo sapiens. (c) The correlation between HNRNPL and PTBP1 in TCGA-PAAD.

To clarify the biological pathways and function of HNRNPL in oncogenesis, GSEA analysis was performed. This analysis revealed that HNRNPL was involved in many crucial pathways and was correlated with cancer. A total of 136 pathways listed in the HNRNPL high-expression group were enriched, including KEGG SPLICEOSOME, KEGG DNA REPLICATION, KEGG P53 SIGNALING PATHWAY, KEGG CELL CYCLE, KEGG BASE EXCISION REPAIR AND KEGG TIGHT JUNCTION, and NES and the* P* values are shown in Table 4 and Figure 4(a).

### 3.7. The Potential Interactors with HNRNPL in the Development of PC

It is suggested that heterogeneous nuclear ribonucleoproteins interact with a multitude of proteins and small nuclear RNAs, forming tight complexes (spliceosome). To identify the tight interaction genes with HNRNPL, we explored the BioGRID database and demonstrated that there were 1717 published interactions for HNRNPL within Homo sapiens.  Figure 4(b) manifested all of the interactors with at least 2 lines of evidence supporting the direct interaction with HNRNPL. We found that PTBP1 was the one with the most evidence. Thus, we examined the correlation of mRNA levels between PTBP1 with HNRNPL in TCGA-PAAD. Interestingly, Pearson test showed that PTBP1 was strongly correlated with HNRNPL (R = 0.59,* P* value < 0.01, Figure 4(c)). Taken together, these results suggest that HNRNPL and PTBP1 might interact with each other in the pathogenicity of pancreatic malignancies.

## 4. Discussion

Spliceosome, which is made up of hundreds of proteins and small nuclear RNAs, contributes significantly to RNA splicing. The HNRNPs family, as a crucial member of the spliceosome, is attracting growing attention with respect to the association with cancer occurrence and progression [21]. There is an ever-expanding body of evidence implicating that the HNRNPs family members are altered in numerous types of tumors, including lung cancer [10], gastric cancer [22], and Merkel cell carcinoma [11]. The expression patterns and prognostic values of HNRNPs in PC have not been clarified.

This manuscript screened out all of the commonly upregulated HNRNPs in TCGA-PAAD and GSE16515 by Venn diagram and determined that* HNRNPA2B1* and* HNRNPL* were upregulated in pancreatic cancer tissues as compared with normal pancreas tissues. Segara Pancreas was employed to provide more evidence with respect to the expression patterns of* HNRNPA2B1* and* HNRNPL*. The* HNRNPA2B1* was not overexpressed in tumor tissues in Segara Pancreas. Previous studies have demonstrated that HNRNPA2B1 is closely related to the invasion and metastases of PC [13, 23] through interacting with KRAS G12V [12]. These results illustrated that HNRNPA2B1 might only be involved in the invasion/metastases of PC, not in the overall occurrence of PC. We further identified that the high expression of* HNRNPL* was a powerful and independent predictor of poor patient outcome, indicating that HNRNPL mRNA level could offer potential value for early diagnosis of PC and tumor monitoring after surgery.

In addition, the mRNA level of HNRNPL was determined to positively associate with tumor invasion and advanced clinical stage. In addition to the mRNA level, HNRNPL protein expression was proven to be correlated with tumor pathologic grade and invasion. These data suggested that the examination of HNRNPL mRNA levels by RT-PCR and protein expression by IHC could both be used as useful tools to identify which PC patients possess risk of cancer invasion. These results underscore an important role of upregulated HNRNPL in the development of PC aggressive nature.

IHC staining indicated that HNRNPL was primarily expressed within the nuclei of pancreatic cancer cells. Harvey et al. reported that the roles of HNRNPs vary depending on their subcellular localization and that most of the HNRNP family members usually keep a nuclear localization signal [24]. Our results manifested that HNRNPL tended to participate in nucleic acid metabolism within the nucleus during the pathogenesis of PC.

We investigated the oncogenic functions of HNRNPL in regulating malignant biological features of PC cells by performing a multitude of experiments in vitro. Our results clearly demonstrated that knockdown of HNRNPL could markedly repress the migration ability and repress the EMT process by downregulating N-cadherin and up-regulating E-cadherin in pancreatic cancer cells. Chao Liu et al. revealed that downregulation of HNRNPL could restrain PANC-1 cell migration, which agreed with our data [25]. In summary, we propose that upregulated expression of HNRNPL, which increases cell invasion and promotes EMT development, results in an enhanced aggressive potential of PC cells.

We also discovered that the proliferation of PC cells was not affected by downregulation of HNRNPL. Depletion of HNRNPL has been reported to significantly suppress cell proliferation of bladder cancer [26] and prostate cancer cells [27]. These data implied that the role of HNRNPL varied among different types of tumors. We further examined the effects of HNRNPL on the pancreatic cancer cell cycle and revealed that downregulation of HNRNPL resulted in G1-phase cell cycle arrest in PC cells.

Given the above lines of evidences that HNRNPL is a potent oncogenic agent in supporting the pathogenic process of PC, we therefore decided to investigate the precise biological mechanisms of HNRNPL in mediating PC cell aggressiveness. HNRNPL, as well as other HNRNP family members, participates in RNA metabolism, as part of the spliceosome, which directly binds to specific sequence element(s) [7, 21]. In addition, HNRNPL was suggested to be involved in the p53 signaling pathway, cell cycle, and tight junctions, which were closely associated with the pathogenesis of PC [28].

Previous studies demonstrated that the HNRNPs family, accompanied by SR proteins and other RNA-binding proteins, played a pivotal role in RNA metabolisms [29]. To discover the potential interactors with HNRNPL, the BioGRID database was utilized to explore all of the genes associated with HNRNPL in Homo sapiens. PTBP1, the gene with most evidence, was identified by BioGRID. Furthermore, it was determined to positively and remarkably associate with the expression of HNRNPL in PC according to TCGA. Additionally, PTBP1 was demonstrated to alter the alternative splicing process of PKM to promote gemcitabine resistance in pancreatic cancer cells [30–32]. These data provided adequate proof for us to speculate that HNRNPL might interact with PTBP1 to together take part in the development of PC.

In conclusion, this study utilizes several cohorts to identify the generally overexpressed HNRNPs, HNRNPL, in PC and demonstrates that downregulation of HNRNPL inhibits pancreatic cancer progression. The study sheds new light on better comprehending the expression patterns and fundamental role of HNRNPs in PC and discovers a potential diagnostic and therapeutic target for PC.

## Figures and Tables

**Table 1:** Cox regression multivariate analysis in TCGA-PAAD.

Group	Num	Hazard ration (95%CI)	*P *value
*Univariate cox model*			

Sex			
Female	80	1	
Male	98	0.809(0.537-1.219)	0.312
Age(year)			
<65	81	1	
≥65	97	1.396(0.918-2.121)	0.118
TNM stage			
I/IIA	49	1	
IIB/III/IV	127	2.050(1.217-3.452)	*0.007∗∗*
Tumor invasion			
T1/2	31	1	
T3/4	145	2.022(1.072-3.815)	*0.030∗*
Histologic grade			
G1	31	1	
G2	95	1.956(1.006-3.803)	*0.048∗*
G3	48	2.622(1.303-5.279)	*0.007∗∗*
G4	2	1.650(0.211-12.885)	0.633
Lymph nodes metastasis			
N0	50	1	
N1	123	2.154(1.282-3.618)	*0.004∗∗*
*HNRNPL* expression			
Low expression	89	1	
High expression	89	1.861(1.222-2.833)	*0.004∗∗*

*Multivariate cox model*			

TNM stage			
I/IIA	49	1	
IIB/III/IV	127	1.772(1.015-3.093)	*0.044∗*
Histologic grade			
G1	31	1	
G2	95	1.667(0.856-3.246)	0.133
G3	48	2.082(1.033-4.197)	*0.040∗*
G4	2	1.300(0.166-10.203)	0.802
*HNRNPL* expression			
Low expression	89	1	
High expression	89	1.171(1.102-2.665)	*0.017∗*

*∗P *< 0.05, *∗∗P *< 0.01.

**Table 2:** The correlation between HNRNPL mRNA level and characteristic features of PC patients in TCGA.

		*HNRNPL*		*χ* ^2^	*P* value
		low expression	high expression		
Age(y)	<65	41	40	0.023	0.880
	≥65	48	49		
Sex	female	49	31	7.356	*0.007∗∗*
	male	40	58		
Tumor invasion	T1	6	1	7.46	*0.044∗*
	T2	16	8		
	T3	65	77		
	T4	1	2		
Grade	G1	19	12	3.399	0.334
	G2	47	48		
	G3	21	27		
	G4	1	1		
TNM stage	I	17	4	10.103	*0.008∗∗*
	II	68	78		
	III	1	3		
	IV	2	3		

*∗P *< 0.05, *∗∗P *< 0.01.

**Table 3:** The correlation between HNRNPL protein expression and characteristic features.

		HNRNPL				*χ* ^2^	*P* value
		-	+	++	+++		
Age(y)	≤60	9	11	2	1	0.915	0.784
	>60	19	14	3	1		
Sex	female	11	11	1	1	1.193	0.764
	male	19	14	4	1		
Tumor invasion	T1	2	3	0	1	9.805	0.432
	T2	24	18	3	0		
	T3	3	4	2	1		
Grade	G2	20	18	1	0	8.404	*0.034∗*
	G3	10	7	4	2		
TNM stage	I	17	9	1	1	10.648	0.045
	II	12	12	4	0		
	IV	1	4	0	1		
Tumor size	≤3.5cm	12	12	1	1	1.529	0.741
	>3.5cm	15	12	4	1		
Nerve infiltration	No	15	17	2	1	2.449	0.490
	Yes	15	8	3	1		
Lymph nodes	No	19	13	3	1	0.619	1.000
	Yes	11	8	2	0		

*∗P *< 0.05.

**Table 4:** Gene set enrichment analysis of HNRNPL in KEGG gene sets.

Name	Size	NES	*P *value	Q value
KEGG_SPLICEOSOME	123	2.35	<0.001	<0.001
KEGG_P53_SIGNALING_PATHWAY	66	2.22	<0.001	<0.001
KEGG_CELL_CYCLE	124	2.15	<0.001	0.002
KEGG_BASE_EXCISION_REPAIR	34	2.04	0.002	0.012
KEGG_PENTOSE_PHOSPHATE_PATHWAY	25	2.01	<0.001	0.015
KEGG_PROTEASOME	43	1.99	<0.001	0.015
KEGG_RNA_DEGRADATION	55	1.98	0.002	0.013
KEGG_NUCLEOTIDE_EXCISION_REPAIR	44	1.96	0.002	0.017
KEGG_PATHOGENIC_ESCHERICHIA_COLI_INFECTION	56	1.95	0.004	0.018
KEGG_DNA_REPLICATION	36	1.93	<0.001	0.022
KEGG_PYRIMIDINE_METABOLISM	95	1.91	0.002	0.025
KEGG_MISMATCH_REPAIR	23	1.90	0.004	0.028
KEGG_ONE_CARBON_POOL_BY_FOLATE	17	1.88	0.008	0.031
KEGG_HOMOLOGOUS_RECOMBINATION	27	1.86	0.004	0.035
KEGG_AMINOACYL_TRNA_BIOSYNTHESIS	41	1.86	0.010	0.033
KEGG_TIGHT_JUNCTION	126	1.84	0.002	0.041

## Data Availability

The data used to support the findings of this study are included within the article.
